# An experimental model to measure the ability of headphones with active noise control to reduce patient’s exposure to noise in an intensive care unit

**DOI:** 10.1186/s40635-017-0162-1

**Published:** 2017-10-17

**Authors:** Stuart Gallacher, Doyo Enki, Sian Stevens, Mark J. Bennett

**Affiliations:** 1 Department of Intensive Care, Derriford Hospital, Plymouth Hospitals NHS Trust, Plymouth, UK; 2 Medical Statistics Group, Plymouth University Peninsula Schools of Medicine and Dentistry, Plymouth, UK; 3 Department of Intensive Care, University Hospital Southampton NHS Foundation Trust, Southampton, UK; 4 Cardiac Intensive Care Unit, Level 6, Derriford Hospital, Plymouth Hospitals NHS Trust, Plymouth, UK

**Keywords:** Noise, Active noise control

## Abstract

**Background:**

Defining the association between excessive noise in intensive care units, sleep disturbance and morbidity, including delirium, is confounded by the difficulty of implementing successful strategies to reduce patient’s exposure to noise. Active noise control devices may prove to be useful adjuncts but there is currently little to quantify their ability to reduce noise in this complex environment.

**Methods:**

Sound meters were embedded in the auditory meatus of three polystyrene model heads with no headphones (control), with headphones alone and with headphones using active noise control and placed in patient bays in a cardiac ICU. Ten days of recording sound levels at a frequency of 1 Hz were performed, and the noise levels in each group were compared using repeated measures MANOVA and subsequent pairwise testing.

**Results:**

Multivariate testing demonstrated that there is a significant difference in the mean noise exposure levels between the three groups (*p* < 0.001). Subsequent pairwise testing between the three groups shows that the reduction in noise is greatest with headphones and active noise control. The mean reduction in noise exposure between the control and this group over 24 h is 6.8 (0.66) dB. The use of active noise control was also associated with a reduction in the exposure to high-intensity sound events over the course of the day.

**Conclusions:**

The use of active noise cancellation, as delivered by noise-cancelling headphones, is associated with a significant reduction in noise exposure in our model of noise exposure in a cardiac ICU. This is the first study to look at the potential effectiveness of active noise control in adult patients in an intensive care environment and shows that active noise control is a candidate technology to reduce noise exposure levels the patients experience during stays on intensive care.

## Background

Excessive noise in the intensive care unit (ICU) environment is thought to be a significant factor in sleep disturbance and that this in turn can result in increased morbidity [1]. Changing the environment [2, 3] and culture [4] in a unit can reduce noise, but these modifications and behaviours are hard to implement and sustain. Patient-specific devices such as earplugs and earphones [5, 6] have been tested but the limited clinical benefit reported may at least in part be due to their lack of efficacy to significantly reduce the noise exposure experienced by the patient. There are no publications quantifying the effectiveness of either of these devices to reduce patient’s exposure to noise in an actual intensive care environment.

ICUs are noisy environments. Mean sound levels are frequently reported in the range of 45–65 dB with peaks above 85 dB commonplace [7]. The World Health Organization (WHO) noise guidelines state noise should not exceed 35 dB during the day with maximum sound level peaks below 40 dB at night in a hospital to ensure a good sleep [8] although the clinical benefit of this approach, or even of a more moderate degree of noise reduction, remains largely untested and without a proven method to reduce noise exposure. Apart from this lower limit of noise exposure, it has been claimed that single-noise events above a sound exposure level of 60 dB are associated with an increased frequency of awakenings [9].

We therefore designed a model of objective noise exposure in a busy cardiac ICU. The aim was to quantify the ability of headphones, either alone or with active noise control (ANC) technology, to reduce the duration and severity of noise exposure above these predefined limits.

## Methods

### Setting

The study was conducted in an open plan 10-bed post-cardiac surgery intensive care unit in a tertiary referral hospital.

### Model

Three identical polystyrene heads, normally used for displaying hats or wigs, were modified to accommodate a sound meter so that the microphone protruded from the ‘auditory meatus’ at an angle to allow a pair of headphones (Bose QuietComfort 15 Acoustic Noise Cancelling Headphones, Bose, Framingham, MA, USA) to be placed. On each occasion, the model was set up in a bed space occupied by a post-cardiac surgery patient where standard treatment was expected without admission or discharge in the following 24-h period.

### Instrumentation and setup

**Fig. 1:**
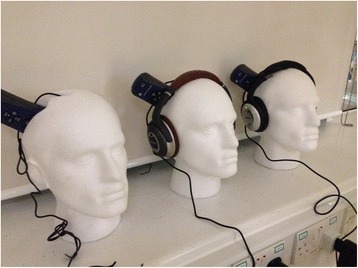
Three polystyrene model heads were placed on a firm shelf close to each other

Three sound level meters: PCE-322A (PCE Instruments, Southampton, UK) were used. These models were set up in the same order by the same investigator on each of the 10 sampling periods. Each microphone was placed as close as was practical to the head of the bed, ventilator and monitoring system. All of the sound meters were calibrated according to the manufacturer’s recommendations. Ten 24-h recording periods between 18:00:00 and 17:59:59 were undertaken on a selection of days of the week to ensure an adequate data capture. Sampling days were selected randomly with a weighting towards weekdays given the predominantly elective caseload. During each 24 h recording period, three measurements occurred simultaneously: control (head only), headphones-off (head and headphones, noise cancellation switched off (NCHoff)) and headphones-on (head and headphones with noise cancellation switched on (NCHon)). The heads were placed on a firm shelf close to each other so that the microphone tip was 2 m behind and equidistant from the ventilator and the patient’s monitoring system (Fig. 1). In the clinical setting, this represents a similar distance from the equipment to the patient’s head.

All recordings were made with ‘A’ frequency-weighting decibel scale as this most closely replicates the ability of the human ear to filter low-frequency noise. Sampling frequency was 1 Hz, with 86,400 recordings made per 24-h period, per recorder. The data were downloaded using the sound level meter datalogger (PCE Instruments) software, and then, uploaded into Excel (Microsoft, Seattle, WA, USA) for analysis.

### Validation of the model

To confirm the validity of our model, a PCE-322A sound level meter was set up without a polystyrene head and another set up as the control described above. Simultaneous recordings from these two were made over a 24-h period. Sampling frequency was 1 Hz. The significance of differences in the data recorded in each group was determined using paired *t* test.

### Statistical analysis

Taking into account the multiple times of measurements made on each day, the hourly data on average noise levels of the three groups were compared using repeated measures multivariate analysis of variance (rmMANOVA). Subsequent pairwise testing was used to further analyse these differences with Bonferroni adjustment for multiple comparisons.

In addition to mean noise levels, we analysed the full combined dataset of 1-s measurements (*n* = 864,000) to measure noise level breaches above 60 dB. Breaches within one full minute of measurement were counted, and the average number per minute in each hour of the day was recorded.

A paired *t* testing for model validation was performed using the Python programming language (Version 2.7.8, Python Software Foundation, Wilmington, DE, USA, https://www.python.org). Repeated measures MANOVA and subsequent pairwise testing was performed using SPSS (IBM SPSS statistics 23, IBM, Armonk, NY, USA). Python graphical library Matplotlib [10] and R statistical programming language (RStudio Version 0.99.903, R Foundation for Statistical Computing, Vienna, Austria) were used for plotting graphs.

## Results

### Model validation

The noise detected within the model was amplified compared with the sound level meter alone (*p* < 0.0001, mean difference 1.9 dB 95% CI 1.855–1.937). This exaggerates the actual noise levels recorded. Importantly, there was no dampening effect of loud sounds or selective sound ‘drop out’ that would change the overall distribution of the data.

### Noise in the intensive care unit

**Fig. 2:**
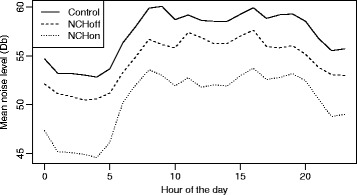
Mean sound level recorded in each hour of the day

**Fig. 3:**
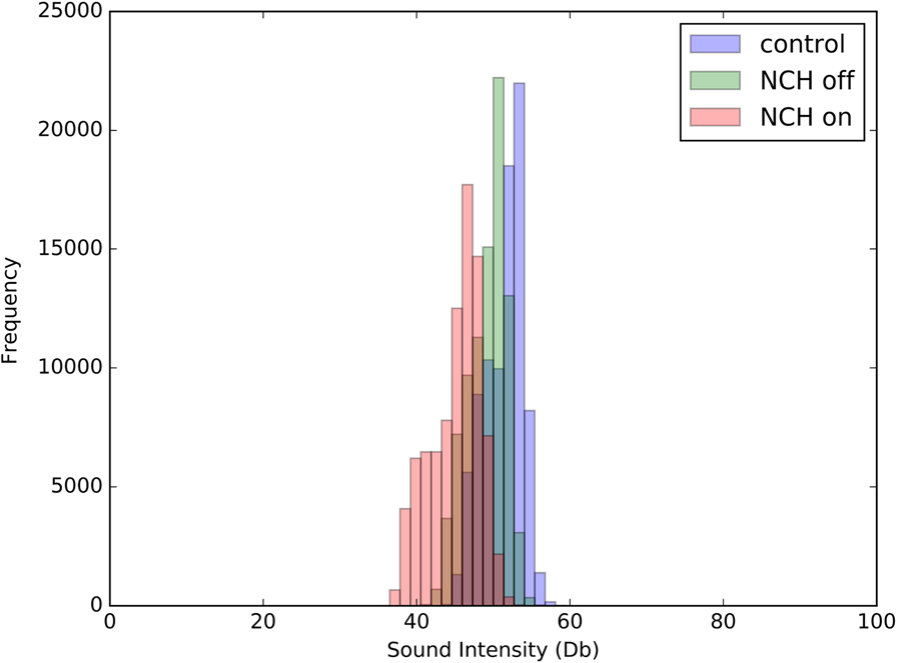
Overlapping histogram of the mean sound intensities

Noise levels demonstrated a diurnal variation with the quietest period between midnight and 05:00 h. The mean sound level recorded in each hour of the day, and in the three groups, over a 24-h period is shown in Fig. 2. An overlapping histogram of the mean sound intensities measured in each of the three groups, at each time point of measurement (i.e. 18:00:00, 18:00:01), over the course of the 10 days of recording is shown in Fig. 3. This demonstrates the same distribution irrespective of group with no selective dropout across the range of sound intensity. No minute in any group fell below 35 dB.

### Noise attenuation by headphones or headphones with active noise control

There was a significant difference in the noise level recorded between groups over time (*p* < 0.001). There was no interaction between the experimental groups and time (Pillai’s Trace *p* = 0.541); therefore, the noise reduction effect, relative to the control group, was consistent over the course of a day. Pairwise comparison between the three experimental groups demonstrated a significant difference between each of the three experimental groups (Table 1). The mean noise levels for the control, NCHoff and NCHon groups are 57.16, 54.49 and 50.36 dB, respectively.

**Table 1:** Summary of pairwise comparison between the three experimental groups

Group comparison	Mean noise difference (standard error)	95% confidence interval for difference	*p* value
Control vs NCHoff	2.67 (.66)	1.00–4.34	.001
Control vs NCHon	6.80 (.66)	5.12–8.47	< .001
NCHoff vs NCHon	4.13 (.66)	2.45–5.80	< .001

**Fig. 4:**
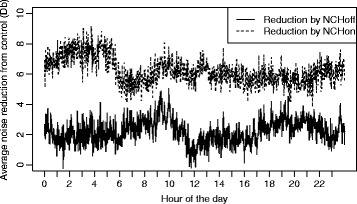
The per-minute variation in the reduction of noise levels over the course of 24 h between the three groups

**Fig. 5:**
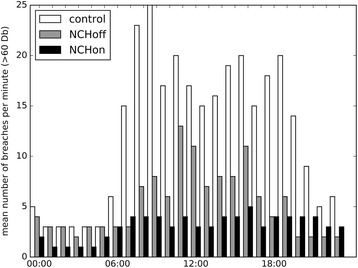
The number of breaches above 60 dB per minute

**Fig. 6:**
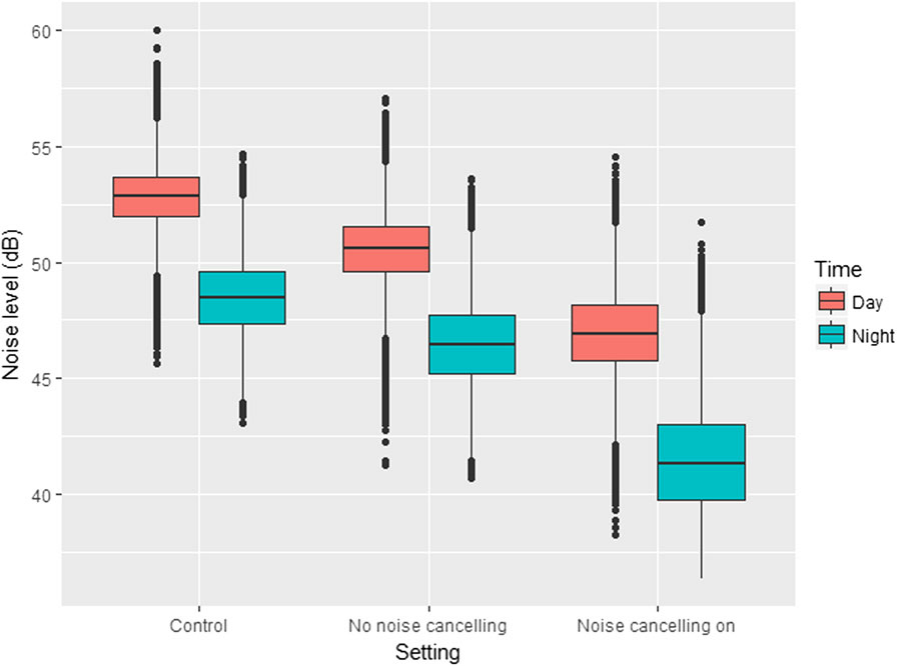
Box plots of the sound levels recorded in each of the three groups during the day and between 2200 and 0600

The per-minute variation in the reduction of noise levels over the course of 24 h between the three groups is shown in Fig. 4. The number of breaches above 60 dB per minute, averaged per hour, across the time period and by group is shown in Fig. 5. There were significantly fewer breaches above this threshold per minute with ANC, but even in this group breaches above 60 dB were not eliminated. Figure 6 shows box plots of the sound levels recorded in each of the three groups during the day and between 2200 and 0600.

## Discussion

We have described a model that permits for the first time quantification of the ability of headphones alone and headphones with ANC to reduce noise experienced in an ICU environment. We have demonstrated that the use of headphones with ANC technology can significantly reduce noise experienced throughout the day. The effect is additive to the reduction in noise exposure achieved by over-ear headphones alone and remains constant over the course of a 24-h period. While the absolute mean reduction over 24 h of 6.8 dB between the NCHon group and our control appears modest, the decibel scale is logarithmic, and this corresponds to a marked reduction in sound energy exposure. Importantly, headphones with ANC also appear to reduce the number of ‘breaches’ of higher intensity sound events, suggesting there may be a ‘smoothing’ of the sound profile of the day as higher intensity sound events are suppressed.

The relative ineffectiveness of headphones alone, and perhaps by inference of earplugs, to reduce noise exposure questions the validity of previous conclusions of the ability of earplugs to alter sleep patterns and other clinical outcomes, including delirium [5, 6].

The only study employing ANC in an ICU sound environment has been used to evaluate the reduction in noise exposure experienced by caregivers rather than patients [11] highlighting the noisy environment, but without benefit to patients. ANC has been investigated to reduce noise experienced during MRI scanning [12]; however, there have been no investigations of its potential in ICU.

ANC was first patented in 1934 by Paul Lueg, a German Physicist [13], and works on the principle of phase opposition; to any given sound, a sound cancellation speaker emits a sound wave of equal amplitude and inverted phase to ‘cancel out’ the emitted sound. This is employed by noise-cancelling headphones in a dynamic fashion to reduce the subjective experience of noise. ANC has been used widely in aviation for many years by pilots to reduce their exposure to noise emitted from engines [14]. Commercially available noise-cancelling devices are now inexpensive, widely available and exist as both over-ear headphones and in-ear earplugs. While this study used over-ear headphones, patients may better tolerate earplugs over longer periods of time, being physically less restrictive while using the same ANC technology.

### Limitations

This work is limited in several important ways. As this is a model, the clinical significance of the reduction in average noise exposure and high intensity noise events cannot be inferred.

Most attempts to address noise in the ICU have concentrated on noise levels, and this has also been the focus of our investigation. However, we acknowledge that the sound spectrum, which is the plotted relationship between frequency and sound level and reverberation time, that is the time taken for a sound to decay after the source has stopped, are important for sound perception, as highlighted by Xie et al. [15]. The increased mean noise recorded when compared with the sound level meter alone may represent an echoic effect of the polystyrene head. There may also be a selective effect of dampening sounds of certain frequencies of noise. As frequency was not recorded, we are unable to rule this out. Additionally, the model design precluded trial of earplugs as a comparison group as the sound meter occupies the auditory meatus. Therefore, only very limited comparison between effectiveness of earplugs and headphones with ANC can be made. While every effort was made to ensure that the model was set up identically on each sampling day, it is possible that small differences in the model setup could result in artefactual differences in the noise levels recorded. We have contacted the manufacturers of the headphones used in this study, and they have unfortunately been unable to supply substantive claims of actual noise reduction levels in either laboratory or real-life environments, including clinical. Finally, while costs of headphones with ANC are falling and re-use of headphones between patients is possible, it is likely that the cost of headphones with ANC will likely exceed that of single use earplugs for the foreseeable future.

## Conclusions

This work is the first to look at ANC on a model of adult patients in an ICU environment. Noise-cancelling headphones are associated with a recorded mean reduction in noise exposure over a 24-h period of 6.8 dB and a reduction in the exposure to high intensity sound events in our model. This effect appears to be constant over the course of a typical day, and although we were unable to reduce noise levels below suggested ‘ideal’ standards, ANC may form an important contributor alongside other measures to achieve this.
